# Supplementary material for: Shotgun Lipidomics for Differential Diagnosis of HPV-Associated Cervix Transformation
Source: Metabolites. 2022 May 31;12(6):503. doi: 10.3390/metabo12060503 (PMC9229224; doi:10.3390/metabo12060503)
Supplement: Supplementary file 1 [file metabolites-12-00503-s001.zip › Supplementary1.pdf]

Supplementary

Shotgun Lipidomics for Differential Diagnosis of HPV-Associated Cervix Transformation

Natalia L. Starodubtseva [1,2,*], Vitaliy V. Chagovets [2], Maria E. Nekrasova [2], Niso M. Nazarova [2], Alisa O. Tokareva [2,3], Olga V. Bourmenskaya [2], Djamilja I. Attoeva [2], Eugenii N. Kukaev [1,2,3], Dmitriy Y. Trofimov [2], Vladimir E. Frankevich [2] and Gennady T. Sukhikh [2,4]

[1] Moscow Institute of Physics and Technology, 141700 Moscow, Russia; e_kukaev@oparina4.ru

[2] National Medical Research Center for Obstetrics Gynecology and Perinatology named after Academician V.I., Kulakov of the Ministry of Healthcare of Russian Federation, 117997 Moscow, Russia; v_chagovets@oparina4.ru (V.V.C.); m_nekrasova@oparina4.ru (M.E.N.); n_nazarova@oparina4.ru (N.M.N.); alisa.tokareva@phystech.edu (A.O.T.); o_bourmenskaya@oparina4.ru (O.V.B.); d_attoeva@oparina4.ru (D.I.A.); d_trofimov@oparina4.ru (D.Y.T.); vfrankevich@gmail.com (V.E.F.); g_sukhikh@oparina4.ru (G.T.S.)

[3] V.L. Talrose Institute for Energy Problems of Chemical Physics, Russia Academy of Sciences,
119991 Moscow, Russia

[4] Department of Obstetrics, Gynecology, Perinatology and Reproductology, First Moscow State Medical University named after I.M. Sechenov, 119991 Moscow, Russia

* Correspondence: n.starodub@phystech.edu

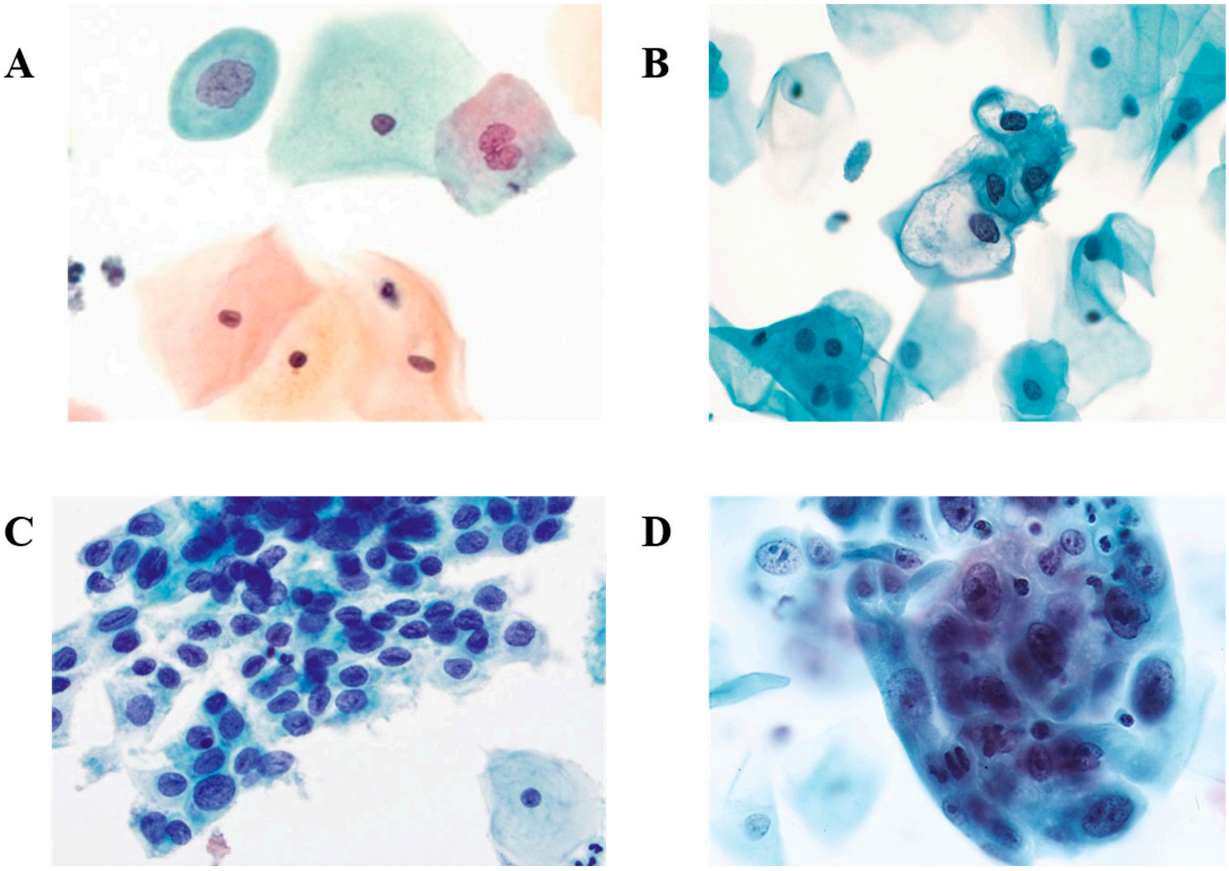

Figure S1. Cytology: (**A**) chronic cervicitis with HPV infection; (**B**) LSIL, (**C**) HSIL and (**D**) cervical cancer.

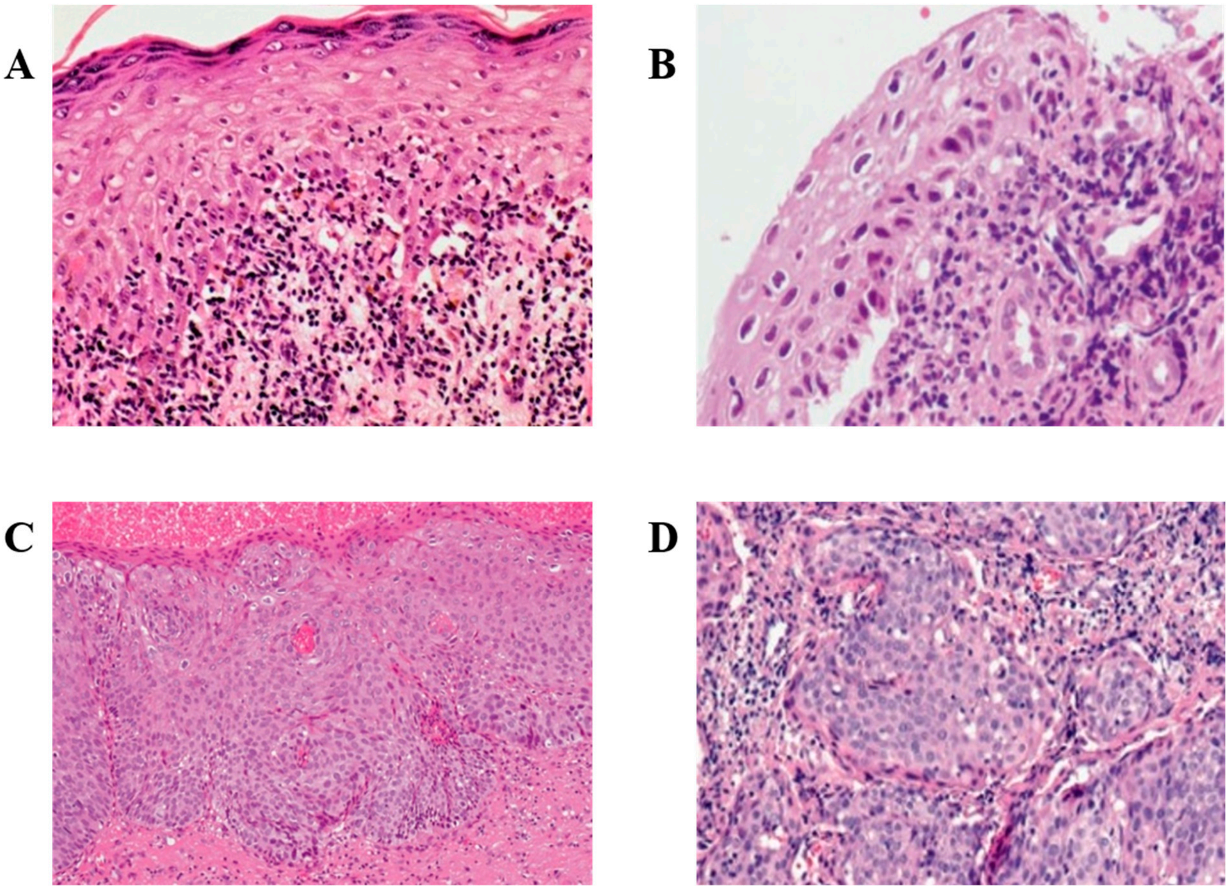

Figure S2. Hystology: (**A**) chronic cervicitis with HPV infection; (**B**) LSIL, (**C**) HSIL and (**D**) cervical cancer.

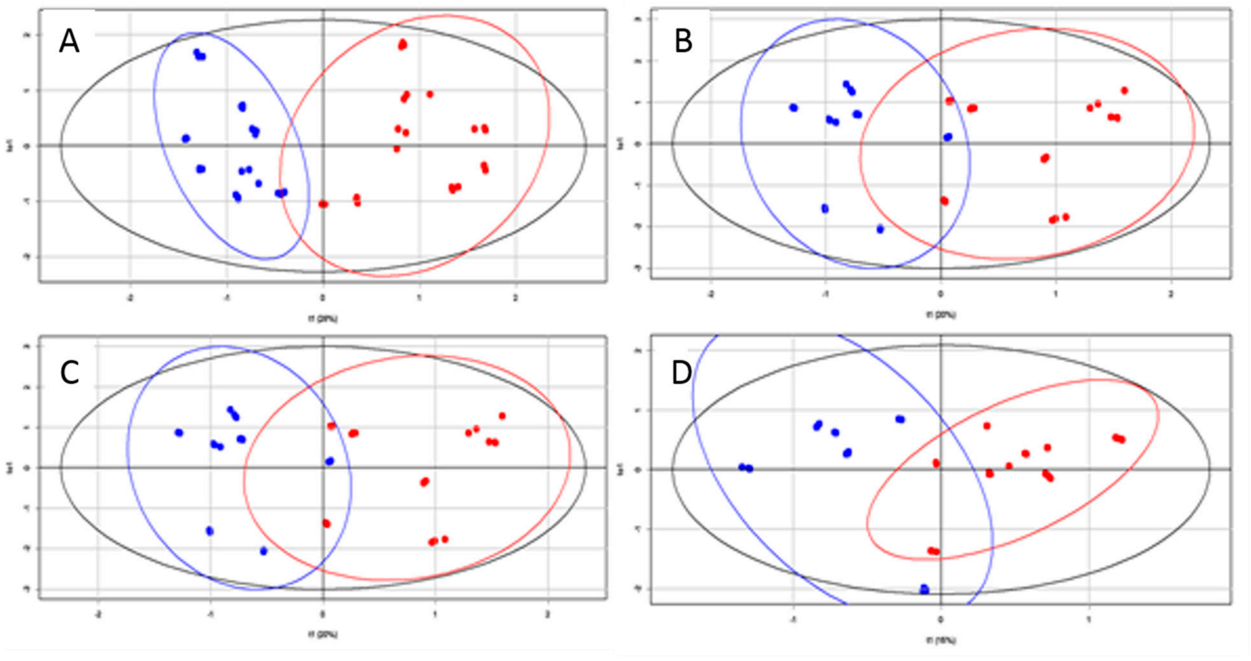

Figure S3. OPLS-DA score plots of ESI-MS data for damaged and surrounding tissues from patients with (**A**) chronic cervicitis; (**B**) LSIL; (**C**)

HSIL; (**D**) SCC. Blue dots correspond to surrounding tissues, red dots - to surrounding tissues.

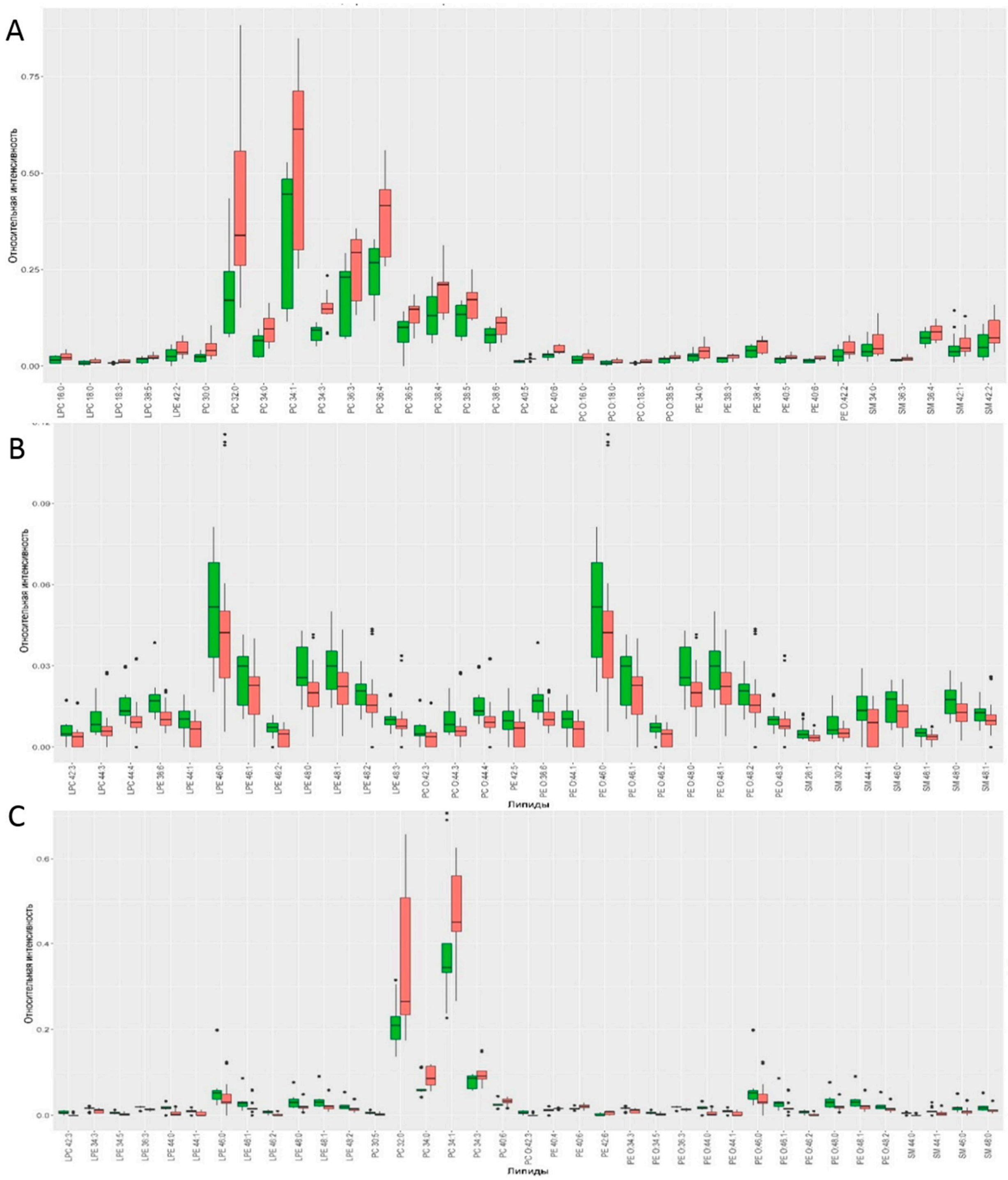

Figure S4. Lipids with mail contribution in the OPLS-DA models for the differentiation of damaged from surrounding tissues: (**A**) LSIL, (**B**) HSIL, (**C**) Cervical cancer. The surrounding tissues are marked in green, the damaged tissues are in red.
